# Immunohistological analysis of extracted anterior cruciate ligament graft impinged against posterior cruciate ligament

**DOI:** 10.1186/1758-2555-3-26

**Published:** 2011-11-02

**Authors:** So Kato, Atsushi Fukai, Hideki Takeda, Shuji Taketomi, Shuichi Nakayama, Jinso Hirota, Kohei Nakajima, Kozo Nakamura, Takumi Nakagawa

**Affiliations:** 1 The Department of Orthopaedic Surgery, Faculty of Medicine, The University of Tokyo, 7-3-1 Hongo, Bunkyo-ku, Tokyo, 113-0033, Japan

## Abstract

A young female athlete suffered from the residual instability of the knee after anterior cruciate ligament (ACL) reconstruction with hamstring autograft. The 3-dimensional (3-D) CT scan showed the "high noon" positioning of the primary femoral bone tunnel. The revision surgery with anatomic double-bundle technique was performed two years after the primary surgery and the femoral tunnels were created with the assistance of the 3-D fluoroscopy-based navigation. An arthroscopic examination confirmed the ACL graft impingement against posterior cruciate ligament (PCL) when the knee was deeply flexed. The histological analysis of the resected primary ACL graft showed local inflammatory infiltration, enhanced synovial coverage and vascularization at the impinged site. The enhanced expression of vascular endothelial growth factor (VEGF) at the impinged area when compared with non-impinged area was observed on immunohistochemical analysis. Abnormal mechanical stress by the impingement against PCL might have induced chronic inflammation and VEGF overexpression.

## Background

Currently, it is well known that roof impingement or posterior cruciate ligament (PCL) impingement of anterior cruciate ligament (ACL) graft can adversely affect the postoperative result, including range of motion (ROM) and knee stability [1]. Positioning both femoral and tibial bone tunnel apertures inside the ACL insertion is essential to create impinge-free ACL graft [2]. Although impingement is supposed to cause graft failure, the underlying molecular mechanism remains to be elucidated. Vascular endothelial growth factor (VEGF) is a potent mediator of angiogenesis, which involves activation, migration, and proliferation of endothelial cells in various pathological conditions [3]. In a sheep ACL reconstruction model, VEGF treated semitendinosus graft showed decreased graft stiffness, although it had a remarkable increase in synovial tissue with hypervascularity [4]. We experienced a case in which malpositioning of femoral tunnel in primary ACL reconstruction caused PCL impingement and graft loosening as a consequence. At the revision ACL surgery, the stretched-out graft was extracted and immunohistological analysis was performed both at the impinged site and non-impinged site to clarify possible molecular changes induced by increased strain.

## Case presentation

A 14-year-old female athlete sustained ACL injury by twisting her left knee while playing football. She underwent single-bundle ACL reconstruction with hamstring autograft in another hospital. After the operation, she suffered from loss of flexion of the reconstructed knee and vigorous physical therapy was performed to regain full range of motion. Nine months later, the range of motion of the operated knee got fully recovered. She got back to previous athletic activity and started playing football again. Although there was no episode of major trauma, she continued to feel unstable on her reconstructed knee soon after returning to the previous sports activity. The hydroarthrosis of the operated knee also recurred. She was referred to our hospital calling for second opinion. The physical examination showed positive Lachman test, anterior drawers test and pivot shift test. KT-2000 knee arthrometer at 134 N revealed 6.5 mm of side-to-side difference. Flexion was 150° and 5° of hyperextension was noted, which was equal to the contralateral side. The 3-dimensional (3-D) CT image of her left femur showed "high noon" malpositioning of the femoral tunnel. The MRI images showed the continual ACL graft with different orientation from native ACL. The intra-articular midsubstance portion of the graft exhibited increased signal intensity although individual fibers seem to be intact. Due to apparent instability of her left knee, she was diagnosed as ACL graft failure and was scheduled to undergo ACL revision surgery.

**Figure 1:**
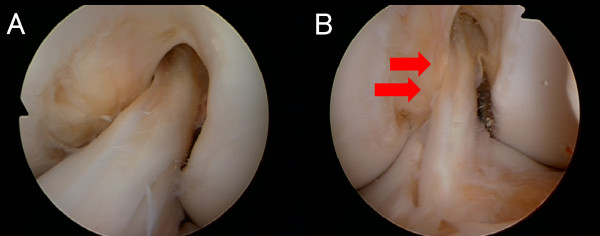
**Arthroscopic views showing ACL graft impingement against PCL**. (A) Arthroscopic view through lateral portal showing "high noon" positioning of the femoral aperture. (B) Arthroscopic view through medial portal at the knee flexion showing PCL impingement the graft (red arrows).

**Figure 2:**
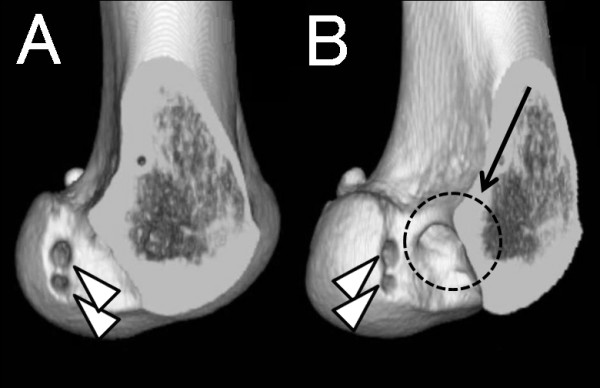
**Postoperative 3-D CT image after the anatomic double-bundle revision ACL reconstruction**. From the medial view (A) and posteromedial view (B). Original high noon tunnel (arrow) and two new anatomically placed femoral tunnels for AM and PL bundles (arrowheads) are shown.

During the revision surgery, ACL graft impingement against PCL was noticed when the knee was deeply flexed on the arthroscopic observation (Figure 1B). The nerve hook palpation confirmed the previous graft loosening as a whole, although there was no macroscopic graft rupture at the impinged site (Figure 1B). The loosened ACL graft was extracted *en bloc *for histological analysis. Anatomic double-socket revision ACL reconstruction was performed using intraoperative 3-D fluoroscopy-based navigation system [5]. While placing the guide wire for the femoral tunnels, the navigation enabled to monitor the original non-anatomic tunnel. The double femoral tunnels were placed anatomically without any communication with the primary tunnel (Figure 2). For the tibial tunnel, the original tunnel was used and the double ACL grafts using contralateral semitendinous and gracilis tendons were placed. Under arthroscopic observation, the revised ACL graft showed no roof or PCL impingement through the range of motion of the knee. Nine months later, she returned to football at the same level before the injury. At two years after the revision, she does not complain any knee instability. ROM was 0°-0°-150° and KT-2000 at 134 N indicated 1 mm side-to-side difference.

**Figure 3:**
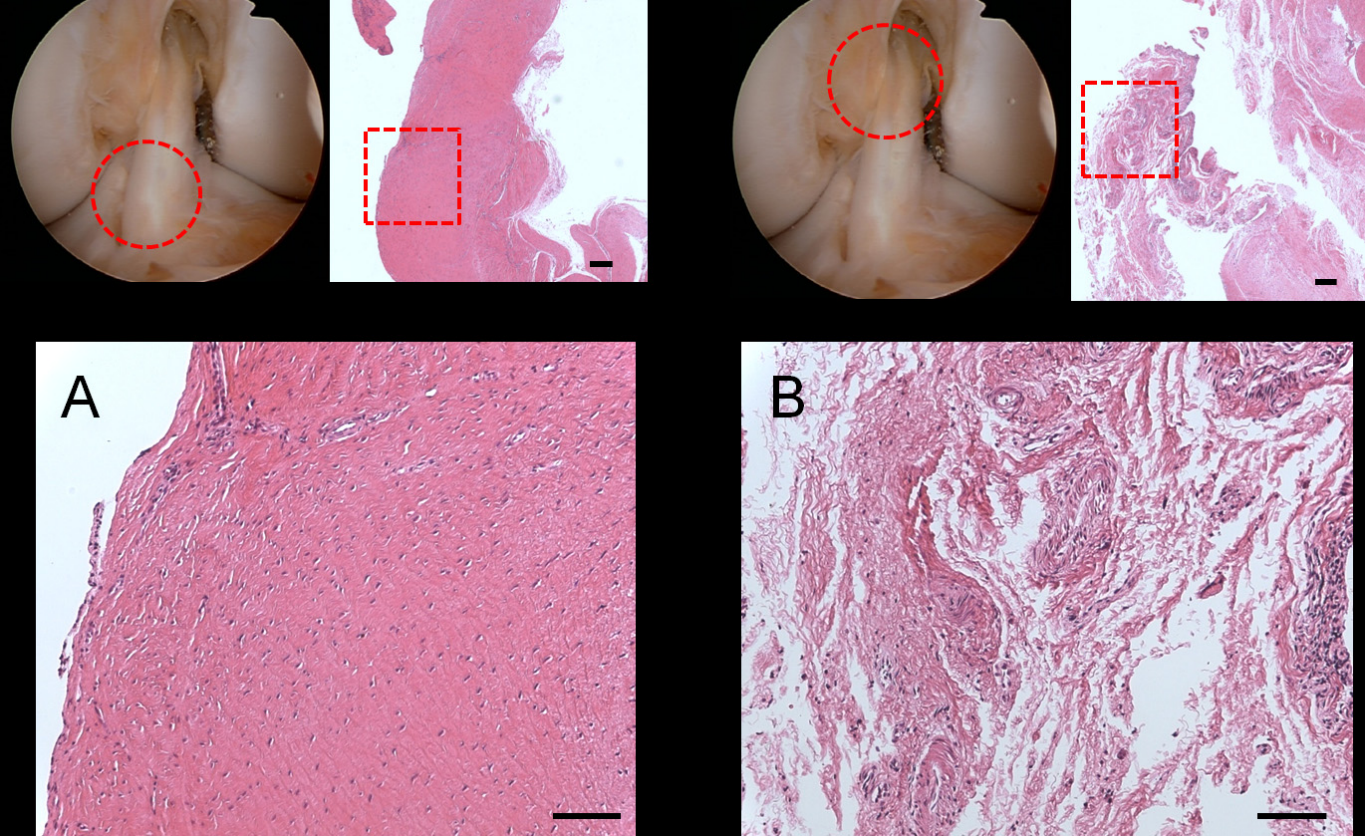
**Photomicrographs of sections of the extracted graft. (Hematoxyline and eosin stains)**. (A) The distal non-impinged part of the ACL graft showing normal synovial coverage and regular pattern of the collagen fibers. (Superior left: arthroscopic view, Superior right: original magnification × 25, Scale bar = 200 μm, Inferior: original magnification × 100, Scale bar = 100 μm) (B) The proximal impinged part against PCL showing enhanced vascularization and proliferation of the overlying synovium. The sections showed irregularity of the collagen fibers and scattered hyaline degeneration of the graft. (Superior left: arthroscopic view, Superior right: original magnification × 25, Scale bar = 200 μm, Inferior: original magnification × 100, Scale bar = 100 μm).

**Figure 4:**
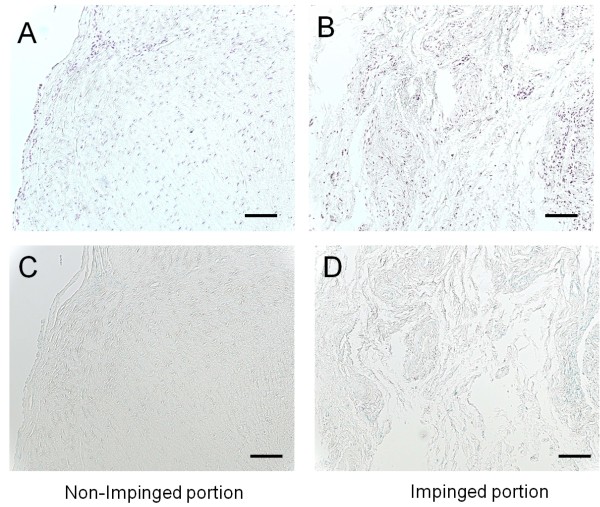
**Immunolocalization of VEGF in the non-impinged portion and impinged portion of the extracted ACL graft**. (**A, B**) Immunohistological stain with anti-VEGF antibody showing the enhanced expression of VEGF at the impinged site of the graft compared with the non-impinged part. (A, B: original magnification × 100, Scale bar = 100 μm) (**C, D**) As negative controls, anti-ADAMTS-5 antibody was substituted for primary antibody. (C, D: original magnification × 100, Scale bar = 100 μm) VEGF = vasucular endothelial growth factor; ADAMTS-5 = A Disintegrin and Metalloproteinase with ThromboSpondin mortif -5.

Histological analysis of the distal part of extracted ACL graft, which was not impinged, showed good synovial coverage and moderate vascularization (Figure 3A). In the impinged proximal part, scattered hyaline degeneration of the graft and abnormal growth of the overlying synovium was observed. Irregularity of the collagen fibers was also seen. In addition, vascularization and inflammatory cell infiltration were remarkably enhanced in the impinged area (Figure 3B). Immunohistological stain with anti-VEGF antibody (Santa Cruz Biotechnology, CA, U.S.A.) showed the enhanced expression of VEGF at the impinged site of the graft compared with the non-impinged part (Figure 4A &4B). On the other hand, immunohistochemical evaluation with anti-ADAMTS-5 antibody and purified human immunoglobulins showed no enhanced expression both on the impinged site and non-impinged portion (Figure 4C &4D).

## Discussion

The most important predictor of clinical outcome in ACL reconstruction is tunnel placement [2]. In this case, suboptimal femoral tunnel positioning, so-called high noon positioning, resulted in PCL impingement of the ACL graft, which was confirmed by direct visualization with arthroscopy. In the early phase after the primary reconstruction, the patient suffered from loss of flexion. Once the operated knee restored full range of motion, she reported functional instability with sports. Based on the clinical findings of this case, the malpositioning of the primary femoral tunnel caused the PCL impingement and ACL graft failure as a result, which led to the functional instability during sports activity. Although PCL impingement is known to cause undesirable clinical consequences of motion loss and instability as shown in our case [1], the underlying molecular mechanism of graft failure remains to be elucidated. The clinically stretched out graft in this case was extracted *en bloc *at the revision surgery and the histological analysis including immunohistochemical stain was performed.

It has been reported that ACL graft undergoes graft maturation including synovial coverage and subsequent revascularization after the reconstruction, which is called "ligamentization" process [6]. In the present case, the primary ACL reconstruction was performed two years before the extraction of the graft. Histological analysis of the non-impinged site of the graft showed good synovial coverage and regular pattern of the underlying collagen fibers. Based on this observation, the non-impinged site of the extracted graft underwent satisfactory graft maturation. On the other hand, the histological observation of the impinged area showed scattered hyaline degeneration and irregularity of the collagen fibers. In addition, stronger neovascularization change and inflammatory cells infiltration associated with abnormal growth of the overlying synovium was observed. Enhanced expression of VEGF, a potent mediator of angiogenesis, was observed in the impinged portion of the graft on histochemical stain analysis when compared with the non-impinged portion. VEGF expression is known to be stimulated by hypoxia stress in solid tumors [3]. Thus, it is postulated that local ischemia due to abnormal mechanical stress at the impinged site might have induced the expression of VEGF by fibroblasts or vascular endothelial cells. Tohyama et al. reported that VEGF expression is increased at the early stage after the ACL reconstruction and then is gradually reduced during "ligamentization" process in a rabbit model [4]. They also reported that VEGF application to the sheep ACL graft strongly induced vascularization and decreased the stiffness of the graft. On the arthroscopic evaluation in this case, the ACL graft impingement against the PCL at deep flexion of the knee was noticed. Therefore, it is speculated that the unphysiological stress by PCL impingement produced intermittent local hypoxia of the impinged graft. This localized hypoxia stress might have induced overexpression of VEGF, which is thought to be one of the serial reactions to the graft impingement. VEGF is also reported to induce matrix metalloproteinase (MMP) expression, which cause the degradation of collagen fibers [3]. However, it still remains to be elucidated whether enhanced expression of VEGF at the impinged portion of the graft induces MMP expression and regional degeneration of the graft. In the future, further investigation including expression of MMP would be needed to clarify the molecular mechanism underlying the ACL graft failure caused by impingement.

In the present case, the enhanced vascularization and inflammatory infiltration, as well as VEGF overexpression, was observed in the impinged ACL graft against PCL. These findings can be interpreted as the graft reaction to unphysiological stress caused by impingement. There is possibility that VEGF expression can be the potential marker of the chronic unphysiological mechanical stress on the graft. Clinically, surgeons must make best effort to perform impinge-free ACL reconstruction surgery.

## Consent

Written informed consent was obtained from the patient and the patient's parents for publication of this case report.

## Competing interests

The authors declare that they have no competing interests.

## Authors' contributions

All authors co-wrote the paper and discussed the results for the manuscript preparation. All authors have read and approved the final manuscript.
